# Perceptions of Ethical and Legal Challenges in Artificial Intelligence-Assisted Implant Dentistry Among Dentists: A Cross-Sectional Survey

**DOI:** 10.7759/cureus.115187

**Published:** 2026-08-25

**Authors:** Sindhuja S., Arundhati Singh, Keerthana Chandrasekaran, Barun Kumar, Ifath Nazia Ghori, Sathyapriya Lakshmanan B.

**Affiliations:** 1 Department of Oral and Maxillofacial Surgery, Bharath Institute of Higher Education and Research, Bharath University, Chennai, IND; 2 Department of Oral and Maxillofacial Surgery, Hazaribag College of Dental Sciences and Hospital, Hazaribagh, IND; 3 Department of Technical Writer for Women's Health Products, Hologic, Inc., Marlborough, USA; 4 Department of Oral and Maxillofacial Surgery, Bharati Vidyapeeth (Deemed to Be University) Dental College and Hospital, Navi Mumbai, IND; 5 Department of Computer Science, Jazan University, Jazan, SAU; 6 Department of Anatomy, Bharath Institute of Higher Education and Research, Bharath University, Chennai, IND

**Keywords:** artificial intelligence, dental implants, dentists, ethics, informed consent

## Abstract

Introduction: Artificial intelligence (AI) is being increasingly incorporated into implant dentistry for diagnostic assessment, treatment planning, and clinical decision support. However, its growing clinical application raises concerns regarding patient privacy, informed consent, transparency, accountability, and professional responsibility. The present study aimed to assess dentists' perceptions of the ethical and legal challenges associated with AI-assisted implant dentistry and evaluate their relationship with AI knowledge and professional characteristics.

Materials and methods: Dental professionals participated in this cross-sectional questionnaire-based survey from March to August 2022. A validated survey was employed to evaluate professional and demographic traits, AI expertise, and ethical and legal attitudes. The 10 ethical and legal assertions were graded on a 5-point Likert scale, and the 10-item AI knowledge part was scored from 0 to 10, for a total score of 0-40. Cronbach's α = 0.86 indicated that the questionnaire had acceptable internal consistency. Descriptive statistics, multiple linear regression, one-way analysis of variance (ANOVA), and Pearson's correlation were used to examine the data; p < 0.05 was used to determine statistical significance.

Results: Of 138 dentists approached, 130 participated, yielding a response rate of 94.2%. The participants' mean age was 42.3 ± 7.1 years, and their mean clinical experience was 15.6 ± 5.8 years. The sample included 71 (54.6%) male dentists and 59 (45.4%) female dentists, of whom 50 (38.5%) reported previous AI exposure. The mean AI knowledge score was 5.8 ± 2.4, and the mean ethical and legal score was 27.6 ± 5.6. AI knowledge showed a significant negative correlation with ethical and legal concern (r = -0.482, p < 0.01). Multiple regression explained 37.5% of the variance in ethical and legal concern (R^2^ = 0.375), with AI knowledge emerging as the strongest independent predictor (β = -0.48, p < 0.001), followed by consultancy practice (β = -0.26, p < 0.001), and previous AI exposure (β = -0.22, p < 0.001). The predicted ethical and legal concern scores progressively decreased from 31.2 in the low knowledge group to 27.4 in the moderate knowledge group and 23.5 in the high knowledge group.

Conclusion: Greater AI knowledge and previous exposure were associated with fewer ethical and legal concerns regarding AI-assisted implant dentistry. Structured AI education, appropriate regulatory frameworks, data protection, and clear professional accountability are essential for integrating AI into implant practice.

## Introduction

Artificial intelligence (AI) has become a rapidly advancing technology in contemporary healthcare with considerable potential to enhance diagnostic precision, treatment planning, and clinical decision-making. The integration of AI in dentistry has grown substantially, with applications spanning multiple disciplines, including oral radiology, orthodontics, prosthodontics, periodontics, endodontics, and implant dentistry [1,2]. AI-powered systems can analyze cone beam computed tomography (CBCT) scans, identify anatomical landmarks, evaluate bone quality and quantity, detect pathological changes, and assist clinicians in selecting appropriate implant dimensions and positions [3]. These technological advancements have the potential to improve treatment efficiency, reduce human error, and support more predictable clinical outcomes [4]. However, despite these benefits, the integration of AI into implant dentistry has also introduced important ethical and legal considerations that require careful evaluation [4,5].

The increasing reliance on AI-assisted decision support systems raises concerns regarding patient privacy, data security, algorithmic bias, transparency of AI recommendations, informed consent, and accountability for treatment outcomes [6]. Because AI systems are frequently trained using large volumes of clinical and radiographic data, ensuring confidentiality and compliance with ethical standards has become a major challenge [7]. Furthermore, many AI algorithms operate as "black box" models, which makes it difficult for clinicians to understand how recommendations are generated. This lack of explainability may reduce trust among practitioners and patients and complicate the assignment of legal responsibility when AI-assisted decisions contribute to treatment failure [8]. Questions also remain regarding whether liability should rest solely with the treating dentist or should be shared with software developers and regulatory authorities.

Although several studies have investigated the technical performance of AI in implant dentistry, relatively limited evidence is available regarding dentists' perceptions of its ethical and legal implications, particularly in developing countries, where AI adoption is still evolving [5,9]. Understanding these perceptions is essential for developing educational initiatives, establishing professional guidelines, and formulating regulatory policies that encourage safe and responsible implementation of AI in clinical practice. Insights from practicing dentists can help identify barriers to AI adoption and inform strategies to improve clinician confidence, while safeguarding patient welfare.

This cross-sectional survey aimed to assess dentists' ethical and legal perceptions regarding AI-assisted implant dentistry. The primary objective was to evaluate dentists' perceptions of issues including patient consent, data privacy, legal responsibility, transparency, and regulation of AI systems. The secondary objectives were to assess AI knowledge and determine its association with ethical and legal perception scores, as well as to examine the association of these scores with relevant demographic and professional characteristics. We hypothesized that greater AI knowledge and previous exposure to AI would be associated with lower levels of ethical and legal concern regarding AI-assisted implant dentistry.

## Materials and methods

Study design and setting

The Department of Oral and Maxillofacial Surgery at Bharati Vidyapeeth (Deemed to Be University) Dental College and Hospital in Navi Mumbai, Maharashtra, India, conducted this cross-sectional, questionnaire-based study between March and August 2022. Participants in this study were dental professionals employed in various clinical and academic settings. All respondents' privacy and identities were protected throughout the study, and participation was entirely voluntary. The study protocol was carried out in compliance with the ethical guidelines of the Declaration of Helsinki (2013 revision) and was authorized by the Institutional Ethics Committee (reference number: BVDUDC&H-IEC-295/2022, dated February 18, 2022).

Eligibility criteria

Registered dental professionals with a BDS or MDS qualification and actively engaged in clinical practice, academics, hospital-based practice, private practice, or consultancy were considered eligible for participation. Dentists were required to have a minimum of five years of professional experience and to provide informed consent for participation. Dental professionals from hospital-based, private clinical, consultancy, and institutional/academic settings were included to ensure representation of different practice environments. Undergraduate dental students, dentists with less than five years of professional experience, individuals unwilling to participate, and respondents who submitted incomplete or duplicate questionnaires were excluded from the final analysis.

Sample size estimation

G*Power software (version 3.1.9.7; Heinrich-Heine-Universität Düsseldorf, Düsseldorf, Germany) was used to determine the necessary sample size for multiple linear regression using the fixed model, R^2^ deviation from zero method. The computation took into account seven predictor variables, a two-sided significance threshold (α) of 0.05, statistical power (1-β) of 80%, and an expected effect size of Cohen's f^2^ = 0.14 [10]. The minimum required sample size was 110 participants. To compensate for an anticipated non-response or incomplete response rate of approximately 25%, the target sample was increased to 138 participants.

Development and validation of the questionnaire

A structured questionnaire was developed specifically for the study, following a review of the literature available at the time of study planning concerning applications of AI in dentistry and implantology and the associated ethical, legal, privacy, accountability, and professional considerations. The questionnaire was developed by a multidisciplinary team comprising oral and maxillofacial surgeons from different dental colleges and a specialist with expertise in AI. Oral and maxillofacial surgeons contributed to the development and assessment of items related to the clinical applications of AI, implant treatment planning, identification of anatomical structures, surgical decision-making, professional responsibility, and potential implications for implant practice. The AI specialist reviewed items concerning AI functionality, algorithmic performance, data requirements, transparency, data security, and the limitations of AI-based decision support systems.

The initial questionnaire was independently reviewed by an expert team for relevance, clarity, comprehensibility, simplicity, and appropriateness of the objectives of the study. The experts also evaluated whether the items adequately represented the domains of AI knowledge and ethical and legal perceptions. Items considered ambiguous, repetitive, or technically difficult were modified following discussion and consensus among panel members. This expert review forms the basis for establishing the content validity of the questionnaire.

Pilot testing and reliability

The preliminary questionnaire was pilot-tested among a small number of dentists who met the research eligibility requirements prior to the start of the main survey. The final study sample did not include participants in the pilot test. A pilot survey was undertaken to assess the clarity and comprehensibility of individual items, appropriateness of the response options, logical sequence of questions, ease of completion, and the approximate time required to complete the questionnaire. Feedback obtained during pilot testing was reviewed by the research team, and minor modifications in wording and presentation were incorporated where required. Cronbach's α was used to evaluate the questionnaire's internal consistency once it was finalized. Good internal consistency and reliability were shown by the overall Cronbach's α coefficient of 0.86. After the study instrument was finalized, no other changes were made.

Questionnaire structure

The final questionnaire comprised three components: demographic and professional characteristics, AI knowledge, and ethical and legal perceptions (see Appendices). The first component recorded demographic and professional characteristics including age, sex, years of professional experience, practice type, and previous exposure to AI. Practice type was categorized as hospital-based, private clinic, consultancy, or institutional/academic. Previous exposure to AI was recorded as a dichotomous response (yes or no).

The second component assessed AI knowledge using 10 questions. These items evaluated participants' knowledge regarding AI-based detection of dental conditions on radiographs, AI-assisted determination of implant specifications using CBCT, identification of critical anatomical structures, assessment of bone density and quality, prediction of implant survival and prosthetic outcomes, generation of patient-specific surgical guides, data requirements for training AI algorithms, relationship between AI and professional clinical judgment, dependence of AI performance on the quality of input imaging data, and ability of AI systems to improve through exposure to new data.

The third component consisted of 10 statements assessing ethical and legal perceptions of AI-assisted implant dentistry. The statements addressed the disclosure of AI use to patients, informed consent for the use of clinical and radiographic data, legal responsibility of the treating dentist, potential responsibility of AI developers, transparency and explainability of AI recommendations, protection of patient privacy and confidentiality, algorithmic bias and validation, establishment of regulatory guidelines, preservation of professional clinical judgment, and willingness to incorporate AI into implant treatment planning when adequate ethical safeguards and legal regulations are available.

Scoring criteria

Two principal scores were generated from the questionnaire: the AI knowledge score and ethical and legal concern scores. The AI knowledge section contained 10 questions with dichotomous response options. Each correct response was assigned a score of 1, whereas incorrect responses were assigned a score of 0. Instead of choosing "yes" or "no" for negatively phrased questions, the scientifically correct response was used to determine the score. As a result, the lowest possible AI knowledge score was 0, and the highest possible score was 10, with higher scores denoting a deeper understanding of the uses and constraints of AI in implantology and dentistry. The total knowledge score was categorized as low (0-3), moderate (4-6), or high (7-10) for interpretive analysis.

Ten statements were scored on a 5-point Likert scale in the ethical and legal perception section: 0 = strongly disagree, 1 = disagree, 2 = neither agree nor disagree, 3 = agree, and 4 = strongly agree. The scores for the 10 statements were summed to calculate the overall ethical and legal concern scores. Therefore, the minimum possible total score was 0, and the maximum possible total score was 40. Higher scores represent greater concern regarding the ethical and legal implications of AI-assisted implant dentistry and stronger endorsement of safeguards related to patient consent, privacy, accountability, transparency, algorithm validation, regulation, and preservation of professional clinical judgment. The overall ethical and legal concern scores were treated primarily as continuous variables for the statistical analysis. No arbitrary low, moderate, or high category was assigned to the overall ethical and legal concern score.

Questionnaire distribution and data collection

The finalized questionnaire was converted into an electronic format using Google Forms (Google LLC, Mountain View, CA, USA) and distributed to eligible dental professionals during the study period from January to March 2023. Potential participants were approached through professional networks and contacts involving dental colleges, hospitals, private practitioners, academics, consultants, and professional dental groups. The introductory section of the questionnaire provided information regarding the purpose of the study, voluntary participation, confidentiality of responses, and instructions for completing the questionnaire.

Several measures were adopted to optimize participation and achieve a high response rate. The questionnaire was deliberately kept concise and predominantly consisted of closed-ended questions to reduce respondent burden. The questions were arranged in a logical sequence, clear instructions were provided, and no unnecessary personal identifying information was collected. The participants were allowed to complete the questionnaire at a convenient time during the study period. Non-respondents received polite reminder messages at reasonable intervals without coercion or repeated excessive contact. Participants were also informed that the survey was intended exclusively for academic research, and that individual responses would remain confidential. Responses were screened periodically for completeness. Duplicate submissions and incomplete questionnaires were excluded from statistical analysis.

Outcome measures

The primary outcome was the overall ethical and legal concern score for AI-assisted implant dentistry. The secondary outcomes included the AI knowledge score and its relationship with ethical and legal concern. Associations between ethical and legal concern scores and age, years of professional experience, previous AI exposure, AI knowledge, and practice setting were also assessed. In addition, independent predictors of the overall ethical and legal concern score and predicted ethical and legal concern scores across different levels of AI knowledge were evaluated. Responses were screened for completeness before analysis. Questionnaires with substantial missing responses were excluded from the final analysis, and no statistical imputation of missing data was performed.

Statistical analysis

An analyst (ING) used IBM SPSS Statistics for Windows, Version 26 (Released 2018; IBM Corp., Armonk, New York, United States) to code, enter, and analyze the data. Prior to analysis, data were verified for consistency and completeness. The Shapiro-Wilk test was utilized to assess the distribution of continuous variables. While non-normally distributed data were summarized using median and interquartile range (IQR), normally distributed continuous variables were expressed as mean ± standard deviation (SD). Frequencies and percentages were used to show categorical variables.

Pearson's correlation analysis was performed to examine bivariate relationships among continuous variables, including age, years of professional experience, AI knowledge score, and overall ethical and legal concern scores. Multiple linear regression analysis was subsequently conducted to identify independent predictors of the overall ethical and legal concern score. The predictor variables included age, years of experience, AI knowledge score, previous AI exposure, and practice type, with hospital-based practices considered as the reference category. The results of the regression model were expressed as unstandardized regression coefficients (B), standard errors (SE), standardized beta coefficients (β), t-values, 95% confidence intervals (CIs), and p-values. The overall model performance was evaluated using the coefficient of determination (R^2^) and the adjusted R^2^.

Multicollinearity among the predictor variables was evaluated using variance inflation factors (VIFs). One-way analysis of variance (ANOVA) was used to compare the mean ethical and legal concern scores among hospital-based, private clinics, consultancy, and institutional/academic practice groups, with Bonferroni-adjusted post hoc comparisons planned where appropriate. Predicted ethical and legal concern scores for participants with low (0-3), moderate (4-6), and high (7-10) AI knowledge were calculated using the regression model while holding the remaining covariates at their mean values. All statistical tests were two-sided, and p < 0.05 was considered statistically significant.

## Results

A total of 130 dentists completed the survey out of 138 participants, resulting in a response rate of 94.2%. The mean age of the participants was 42.3 ± 7.1 years, and the mean clinical experience was 15.6 ± 5.8 years. The study included 71 (54.6%) male dentists and 59 (45.4%) female dentists. Regarding practice settings, 38 (29.2%) participants worked in private clinics, 36 (27.7%) in institutional/academic settings, 32 (24.6%) in hospital-based practices, and 24 (18.5%) in consultancy practice. Fifty (38.5%) participants reported previous exposure to AI. The mean AI knowledge score was 5.8 ± 2.4, while the mean ethical and legal concern score was 27.6 ± 5.6 (Table 1).

**Table 1:** Demographic and professional characteristics, AI knowledge, and ethical and legal concern scores of the study participants Data are presented as mean ± SD and range for continuous variables, and as numbers (%) for categorical variables. AI: artificial intelligence; SD: standard deviation

Variable		Value
Age (years)	Mean ± SD	42.3 ± 7.1
	Range	30-55
Years of experience	Mean ± SD	15.6 ± 5.8
	Range	5-25
AI knowledge score	Mean ± SD	5.8 ± 2.4
	Range	0-10
Overall ethical and legal concern score	Mean ± SD	27.6 ± 5.6
	Range	12-38.4
Sex (number (%))	Male	71 (54.6)
	Female	59 (45.4)
Practice type (number (%))	Hospital-based	32 (24.6)
	Private clinic	38 (29.2)
	Consultancy	24 (18.5)
	Institutional/academic	36 (27.7)
Prior AI exposure (number (%))	Yes	50 (38.5)
	No	80 (61.5)

The correlation analysis demonstrated that ethical and legal concern scores were positively correlated with age (r = 0.184, p < 0.01) and years of clinical experience (r = 0.156, p < 0.01). AI knowledge showed a moderate negative correlation with ethical and legal concern scores (r = -0.482, p < 0.01), indicating lower ethical and legal concern among participants with greater AI knowledge (Table 2).

**Table 2:** Correlation of age, professional experience, and AI knowledge with ethical and legal concern scores among study participants Data are presented as Pearson's correlation coefficients (r). *p < 0.05 denotes statistical significance. AI: artificial intelligence

Variable	Age	Experience	AI knowledge	Ethical and legal concern
Age	1.000	0.872*	-0.085	0.184*
Experience	0.872*	1.000	-0.062	0.156*
AI knowledge	-0.085	-0.062	1.000	-0.482*
Ethical and legal concern	0.184*	0.156*	-0.482*	1.000

The multiple linear regression analysis showed a significant association with the overall ethical and legal concern score (R^2^ = 0.375, adjusted R^2^ = 0.354; F = 18.86, p < 0.001). AI knowledge was the strongest independent predictor (β = -0.48, p < 0.001), followed by consultancy practice (β = -0.26, p < 0.001) and previous AI exposure (β = -0.22, p < 0.001). Age also showed a significant positive association (β = 0.19, p = 0.012), whereas years of experience were not independently associated with ethical and legal concern (p = 0.504) (Table 3).

**Table 3:** Factors associated with ethical and legal concern scores regarding AI-assisted implant dentistry using multiple linear regression analysis Model summary: R^2^ = 0.375, adjusted R^2^ = 0.354, F = 18.86, p < 0.001. *Statistically significant at p < 0.05. AI: artificial intelligence, B: unstandardized regression coefficient, β: standardized regression coefficient, CI: confidence interval, SE: standard error, VIF: variance inflation factor

Predictor variable	Unstandardized B	SE	Standardized β	t-value	p-value	95% CI for B	VIF	Rank
Constant	72.15	8.42	-	8.57	<0.001*	55.68 to 88.62	-	-
Age (years)	0.38	0.15	0.19	2.53	0.012*	0.08 to 0.68	4.85	4
Experience (years)	0.12	0.18	0.05	0.67	0.504	-0.23 to 0.47	4.56	7
AI knowledge score	-2.85	0.38	-0.48	-7.50	<0.001*	-3.60 to -2.10	1.08	1
Prior AI exposure (yes)	-5.90	1.62	-0.22	-3.64	<0.001*	-9.10 to -2.70	1.12	3
Practice type (reference: hospital-based) private clinic	-4.25	2.10	-0.15	-2.02	0.045*	-8.38 to -0.12	1.25	5
Consultancy	-8.75	2.35	-0.26	-3.72	<0.001*	-13.38 to -4.12	1.18	2
Institutional/academic	-2.10	2.15	-0.07	-0.98	0.329	-6.34 to 2.14	1.20	6

**Figure 1:**
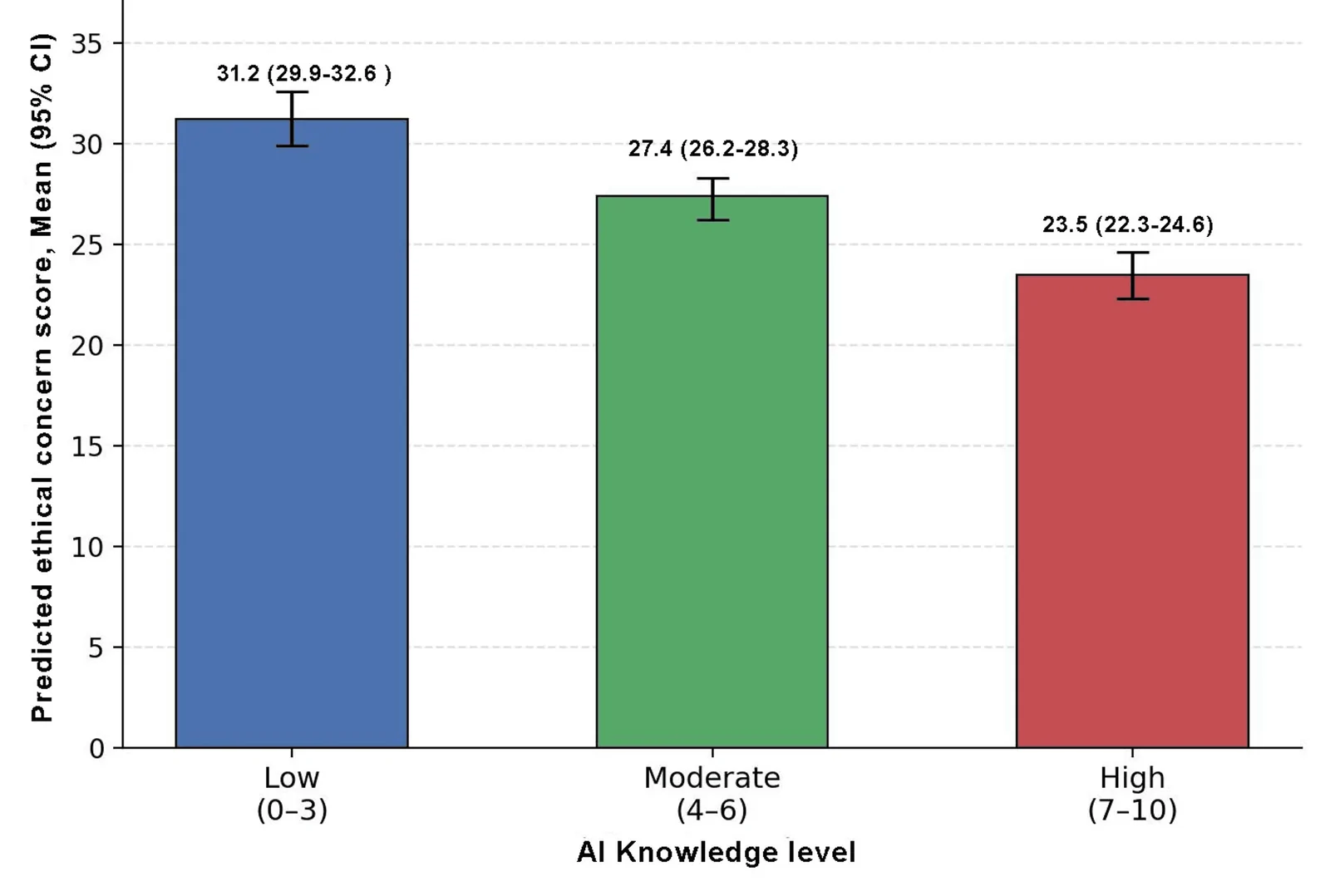
Predicted ethical and legal concern scores across different levels of AI knowledge among study participants Predicted values were derived from the multiple linear regression model with other covariates held at their mean values. AI: artificial intelligence; CI: confidence interval

The predicted ethical and legal concern scores decreased progressively with increasing knowledge of AI. The predicted mean scores were 31.2 (95% CI: 29.9-32.6) for participants with low AI knowledge, 27.4 (95% CI: 26.2-28.3) for those with moderate AI knowledge, and 23.5 (95% CI: 22.3-24.6) for those with high AI knowledge (Figure 1).

Ethical and legal concern scores also varied across practice settings, with the highest mean score observed among hospital-based dentists (28.9 ± 5.5) and the lowest among consultants (25.4 ± 6.0). However, the overall difference between the practice groups was not statistically significant (F = 1.97, p = 0.122) (Table 4).

**Table 4:** Comparison of ethical and legal concern scores among dentists according to practice setting Data are presented as mean ± SD. p < 0.05 was considered statistically significant. CI: confidence interval; SD: standard deviation

Practice type	Ethical and legal concern (mean ± SD)	95% CI of the mean	F value	p-value	Effect size
Hospital-based (n = 32)	28.9 ± 5.5	27.0-30.8	1.97	0.122	0.045
Private clinic (n = 38)	27.2 ± 5.8	25.4-29.0			
Consultancy (n = 24)	25.4 ± 6.0	23.0-27.8			
Institutional/academic (n = 36)	28.1 ± 5.1	26.4-29.8			

## Discussion

This study evaluated dentists' perceptions of the ethical and legal challenges associated with AI-assisted implant dentistry. Overall, the participants demonstrated a moderate level of AI knowledge, together with considerable awareness of the ethical and legal issues associated with its clinical implementation. A key finding was that greater AI knowledge was associated with lower ethical and legal concern, suggesting that familiarity with AI may influence how dental professionals perceive the risks and uncertainties surrounding its use. This finding highlights the importance of AI knowledge in promoting informed, critical, and responsible adoption of emerging technologies in implant dentistry.

AI knowledge emerged as the strongest independent predictor of ethical and legal concern, while previous exposure to AI was also associated with lower levels of concern. Dentists who understand the capabilities and limitations of AI may be better positioned to distinguish realistic ethical risks from concerns arising primarily from unfamiliarity with technology. Similar findings have been reported in previous surveys assessing the knowledge and attitudes toward AI among dental professionals and students. A South Indian study reported considerable uncertainty regarding AI-related ethical issues and demonstrated substantial support for incorporating AI education into the dental curriculum [11]. Such findings reinforce the importance of formal education and structured professional training rather than dependence on informal or incidental exposure to AI technologies [12].

Ethical considerations related to AI in dentistry extend beyond the knowledge and acceptance of technology. Mörch et al. [13], in their scoping review of AI ethics in dentistry, identified several important ethical domains, including privacy, responsibility, equity, prudence, democratic participation, and solidarity. Patient confidentiality, informed consent, accountability, algorithmic transparency, and professional responsibility are particularly relevant when AI systems process clinical and radiographic information for implant treatment planning [14]. These concerns have become increasingly important as AI systems progress from image interpretation to more complex decision support functions.

The present study further demonstrated a consistent trend, whereby increasing AI knowledge was associated with progressively lower ethical and legal concern. This finding supports the possibility that AI knowledge contributes to greater confidence in evaluating AI-assisted clinical systems. More recent evidence from Ozbey et al. [10] similarly demonstrated differences in dentists' awareness of AI-related ethical issues, data security, anonymization, and legal regulations. Their findings emphasized the need for structured professional education regarding data protection, legal responsibilities, and the appropriate clinical use of AI. Collectively, these observations suggest that education should encompass not only the technical applications of AI but also its ethical, legal, and regulatory dimensions [15].

Age and clinical experience showed some association with ethical and legal concern; however, professional experience did not remain an independent predictor when other relevant factors were considered. This suggests that years of clinical practice alone may not adequately prepare dentists to address the ethical challenges of AI. AI knowledge represents a distinct competency that may require specific education irrespective of professional seniority. Previous research has similarly indicated that awareness of AI-related ethical and data security issues may vary across professional and demographic characteristics without following a uniform experience-dependent pattern [10].

The practice environment may also influence the dentists' perceptions. Hospital-based practitioners tended to express greater ethical and legal concern, whereas consultants expressed comparatively lower concerns. Nevertheless, the overall differences between the practice settings were not statistically significant. The independent association observed for consultancy practices may reflect differences in exposure to digital technologies, institutional responsibilities, patient populations, or familiarity with technology-assisted workflows. Therefore, these findings should be interpreted cautiously and further explored in larger studies involving diverse practice environments.

The ethical challenges associated with AI extend beyond its technical performance and encompass fairness, privacy and data protection, responsibility and accountability, transparency and explainability, safety, and robustness. These interconnected concerns highlight the need for responsible governance and appropriate safeguards when AI systems are incorporated into clinical decision-making [15].

From a clinical perspective, AI should serve as a decision support tool that complements rather than replaces professional judgment. Although AI has demonstrated considerable potential in dental imaging, treatment planning, predictive analysis, and workflow automation, its integration into routine practice remains constrained by concerns regarding data privacy, algorithmic bias, regulatory gaps, and inadequate clinical training. Emerging approaches such as explainable AI may improve transparency and facilitate clinicians' understanding of AI-generated recommendations [16]. Furthermore, AI applications frequently rely on sensitive dental data, including radiographs, CBCT scans, clinical photographs, intraoral scans, and electronic health records, thereby emphasizing the need for appropriate de-identification, secure data-sharing practices, and privacy-preserving techniques. Therefore, the successful integration of AI into dentistry requires not only technological advancement but also appropriate professional training, standardized clinical protocols, robust data protection measures, clear regulatory frameworks, and interdisciplinary collaboration [17,18].

The present study had certain limitations. The cross-sectional design does not permit causal conclusions regarding the relationship between AI knowledge and ethical and legal perceptions. The relatively limited sample size and recruitment through professional networks may introduce selection and nonresponse bias and may restrict the generalizability of the findings. Self-reported responses may also be influenced by recall and social desirability biases. Although the questionnaire underwent expert review, pilot testing, and assessment of internal consistency, more extensive psychometric validation, including construct and criterion validity, was not performed. In addition, the composite ethical and legal perception score incorporated several related but conceptually distinct domains, including patient privacy, informed consent, accountability, transparency, regulatory safeguards, and willingness to adopt AI under appropriate safeguards; therefore, the score should be interpreted as an overall perception measure rather than a single homogeneous construct.

Residual confounding related to professional characteristics, practice setting, and previous AI exposure cannot be excluded. Furthermore, perceptions of AI may change rapidly as technologies and regulatory frameworks evolve. The observed association between AI knowledge and ethical and legal perceptions should not be interpreted as evidence that AI education would reduce ethical concerns, as this cannot be established from a cross-sectional design. Future research should include larger multicenter samples representing different geographic regions and dental specialties, employ longitudinal designs, and use more extensively validated instruments to determine whether structured AI education influences ethical and responsible AI perceptions over time. Dental institutions and professional organizations should incorporate AI knowledge, patient privacy, informed consent, algorithmic bias, transparency, accountability, and relevant legal and regulatory principles into undergraduate, postgraduate, and continuing professional education programs.

## Conclusions

Dentists demonstrated moderate AI knowledge and substantial awareness of ethical and legal considerations surrounding AI-assisted implant dentistry. Greater AI knowledge and previous exposure to AI were significantly associated with lower ethical and legal perception scores, with AI knowledge emerging as the strongest independent predictor. These findings support the importance of structured AI education, clear regulatory guidance, protection of patient data, and preservation of professional accountability. AI should complement rather than replace clinical judgment, with its integration into implant dentistry guided by appropriate safeguards related to consent, privacy, transparency, bias, accountability, and regulation.

## Appendices

**Table 5:** Questionnaire used in the study

Demographic details		
S.no.	Question	Response
1	Age	______ Years
2	Years of experience	______ Years
3	Sex	□ Male □ Female
4	Practice type	□ Hospital based □ Private clinic □ Consultancy □ Institutional/academician
5	Prior AI exposure	□ Yes □ No
AI knowledge (0-10 score): Yes = 1, No = 0		
1	AI can be trained to detect dental caries, periapical lesions, and periodontal bone loss on panoramic and intraoral radiographs.	□ Yes □ No
2	AI systems in implant dentistry can automatically determine optimal implant specification based on cone beam computed tomography (CBCT) data.	□ Yes □ No
3	AI-assisted implant planning software can identify critical anatomical structures.	□ Yes □ No
4	AI can be used to predict bone density and bone quality at potential implant sites using preoperative radiographic data.	□ Yes □ No
5	AI algorithms can predict implant survival rates and long-term prosthetic success based on patient-specific risk factors.	□ Yes □ No
6	AI systems can automatically design and generate patient-specific surgical guides for flapless or minimally invasive implant surgery.	□ Yes □ No
7	AI algorithms used for implant planning require large datasets of CBCT scans and clinical outcomes for effective training.	□ Yes □ No
8	AI technology in implant dentistry can completely replace the clinical judgment and surgical skills of an experienced implantologist.	□ Yes □ No
9	The accuracy of AI-assisted implant planning is independent of the quality of the input imaging data.	□ Yes □ No
10	AI systems used in dentistry are static and cannot improve their performance over time with new data.	□ Yes □ No
Ethical score (0-40 score), Likert scale: 0 = strongly disagree, 1 = disagree, 2 = neither agree nor disagree, 3 = agree, 4 = strongly agree		
1	Patients should be informed whenever artificial intelligence is used to assist in dental implant treatment planning.	□ Strongly disagree □ Disagree, □ Neither agree nor disagree □ Agree □ Strongly agree
2	Explicit patient consent should be obtained before using clinical or radiographic data for artificial intelligence-assisted implant treatment planning.	□ Strongly disagree □ Disagree, □ Neither agree nor disagree □ Agree □ Strongly agree
3	The treating dentist should remain legally responsible for all treatment decisions, even when artificial intelligence recommendations are used.	□ Strongly disagree □ Disagree, □ Neither agree nor disagree □ Agree □ Strongly agree
4	Artificial intelligence developers should share legal responsibility if inaccurate recommendations contribute to implant treatment failure.	□ Strongly disagree □ Disagree, □ Neither agree nor disagree □ Agree □ Strongly agree
5	Artificial intelligence systems used in implant dentistry should provide transparent and explainable recommendations rather than acting as a "black box."	□ Strongly disagree □ Disagree, □ Neither agree nor disagree □ Agree □ Strongly agree
6	Protection of patient privacy and confidentiality is a major ethical concern when implementing artificial intelligence in implant dentistry.	□ Strongly disagree □ Disagree, □ Neither agree nor disagree □ Agree □ Strongly agree
7	Artificial intelligence algorithms should be regularly validated to minimize bias and ensure fair treatment recommendations for all patients.	□ Strongly disagree □ Disagree, □ Neither agree nor disagree □ Agree □ Strongly agree
8	National or professional regulatory bodies should establish clear ethical and legal guidelines for the use of artificial intelligence in implant dentistry.	□ Strongly disagree □ Disagree, □ Neither agree nor disagree □ Agree □ Strongly agree
9	Artificial intelligence should be used only as a clinical decision support tool and should not replace the professional judgment of the treating dentist.	□ Strongly disagree □ Disagree, □ Neither agree nor disagree □ Agree □ Strongly agree
10	I would be willing to incorporate artificial intelligence into implant treatment planning if adequate ethical safeguards and legal regulations were in place.	□ Strongly disagree □ Disagree, □ Neither agree nor disagree □ Agree □ Strongly agree
